# Genomic DNA Methylation Changes in NYGGF4-Overexpression 3T3-L1 Adipocytes

**DOI:** 10.3390/ijms131215575

**Published:** 2012-11-22

**Authors:** Lei Yang, Mei-Ling Tong, Xia Chi, Min Zhang, Chun-Mei Zhang, Xi-Rong Guo

**Affiliations:** 1 State Key Laboratory of Reproductive Medicine, Department of Pediatrics, Nanjing Maternity and Child Health Care Hospital Affiliated to Nanjing Medical University, No 123 Tianfei Xiang, Mochou Road, Nanjing 210029, China; E-Mails: yanglei8224982@163.com (L.Y.); kt99cn@yahoo.com.cn (M.-L.T.); chixia2001@yahoo.com.cn (X.C.); ntzhangmin@163.com (M.Z.); 2 Institute of Pediatrics, Nanjing Medical University, No.140 Hanzhong Road, Nanjing 210029, China

**Keywords:** NYGGF4, DNA methylation, insulin resistance, adipocytes

## Abstract

*NYGGF4*, an obesity-related gene, is proposed to be involved in the development of insulin resistance; however, the underlying molecular mechanisms remain unclear. In the present analysis, NimbleGen tiling arrays were used to determine the patterns of genomic DNA methylation at CpG islands and promoters in NYGGF4-overexpression adipocytes. A total of 2352 CpG dinucleotides in 2018 genes and 3490 CpG dinucleotides in 3064 genes were found to be hypermethylated or hypomethylated, respectively, in NYGGF4-overexpression adipocytes. Furthermore, gene ontology (GO) and Kyoto Encyclopedia of Genes and Genomes(KEGG) pathway analysis revealed enrichment of biological processes associated with energy metabolism and signal transduction events, including the peroxisome proliferator-activated receptor gamma (PPARγ) signaling pathway, and mitogen-activated protein kinases(MAPK) and Ras homolog gene family, member A (RhoA) signaling. These data demonstrate that differentially methylated genes are significantly overrepresented in NYGGF4-overexpression adipocytes, providing valuable clues for further exploration of the role of NYGGF4 in insulin sensitivity regulation.

## 1. Introduction

Obesity is a multifactorial disease arising from interactions between environmental factors, genetic predisposition and individual behaviors [1]. Among the different mechanisms which may underlie the interindividual differences in obesity, epigenetic concepts have emerged as a potentially important determinant in recent years [1]. Epigenetics involves the analysis of the inherited changes in gene function that occur independently of alterations in nucleotide sequences [2]. These changes include DNA methylation, covalent histone modifications, chromatin folding and the regulatory action of microRNAs (miRNAs) and polycomb group complexes [3]. Different human genes have been described to be regulated by epigenetic mechanisms in relation to the development of obesity and obesity-related processes [1]. Several investigations have revealed that the promoters of the leptin, peroxisome proliferator-activated receptor gamma (PPARγ) and glucose transporter 4 (GLUT4) genes show characteristics of hypomethylation during adipogenesis [4–6]. Furthermore, clinical research has suggested that the promoter methylation levels of leptin and tumor necrosis factor-alpha (TNF-α) can predict the response to weight loss interventions in obese individuals [7,8]. These remarkable findings have identified epigenetic mechanisms as an attractive therapeutic target for obesity.

Recent advances in epigenetics suggest that nutrition, behavior and metabolic disturbances, as well as other factors, have major effects on epigenetic processes [9]. Recently, Rüegg *et al*. observed that knockout of the estrogen receptor β (*ER-β*) gene led to hypermethylation of a single CpG in the *Glut4* promoter, subsequently reducing transcription of *Glut4* in mouse embryonic fibroblasts, which indicated that specific genes might play important roles in epigenetic processes [10].

NYGGF4 is a newly discovered obesity candidate gene, and our previous studies have demonstrated that overexpression of NYGGF4 can lead to insulin resistance in adipocytes and skeletal muscle cells [11,12]. However, until now, the understanding of how NYGGF4 influences insulin sensitivity has remained fairly rudimentary. The aim of this study was to test whether epigenetic processes are involved in the development of NYGGF4-induced insulin resistance. In the present study, we used a comprehensive methylation profiling technique, termed the “methylated-CpG island recovery assay” or MIRA [13], in conjunction with CpG island and promoter microarrays (MIRA-chip) to characterize the genomic DNA methylation differences in NYGGF4-overexpression adipocytes and control cells. The results of this study provide valuable insight into methylation changes in NYGGF4-overexpression adipocytes and offer clues for further exploration of the mechanisms by which NYGGF4 regulates insulin sensitivity.

## 2. Results and Discussion

### 2.1. The Establishment of Stable 3T3-L1 Preadipocytes Lines Overexpression NYGGF4

**Figure 1:**
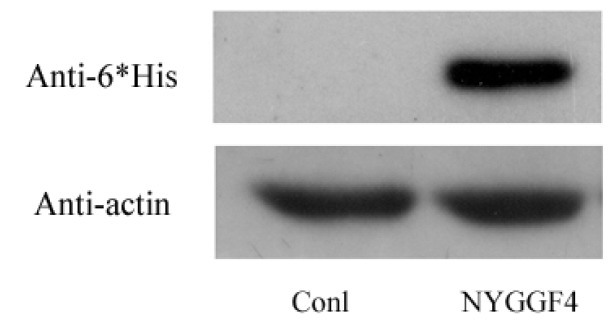
Expression of NYGGF4 in 3T3-L1 preadipocytes transfected with pcDNA3.1Myc/His empty vector (Conl) or NYGGF4-pcDNA3.1Myc/His expression vector (NYGGF4). Total proteins were isolated from the stable cell lines and analyzed the protein expression of NYGGF4 by Western blotting using a tag antibody against 6 × His. β-actin was used as an internal control.

We constructed the NYGGF4-expression sequence which was fused in frame with the *C*-terminal of 6 × His peptide (pcDNA3.1Myc/His/). 3T3-L1 preadipocytes were cultured in a 6-well plate and were transfected with the NYGGF4-expression vector (NYGGF4-pcDNA3.1Myc/His) or the empty vector as control. Twenty four hours after transfection, the cells were digested and were seeded into a 96-well plate (about one cell/well). When the cell adhered, neomycin (G418) was added to the medium (800 μg/mL) to select for stably transfected cells. After two weeks of G418 addition, resistant single colonies were isolated. The protein expression level of NYGGF4 was identified through detection of NYGGF4-6 × His fusion protein level by the Anti-His antibody (Figure 1).

### 2.2. Detection of Differentially Methylated Genes in NYGGF4-Overexpression Adipocytes

To identify the differentially methylated genes in NYGGF4-overexpression adipocytes compared to control adipocytes, we initially classified the CpGs according to their methylation status. A total of 5842 CpG loci covering 5082 genes were classified as differentially methylated in NYGGF4-overexpression adipocytes. A total of 2352 CpG loci (2018 genes) and 3490 CpG loci (3064 genes) were hypermethylated or hypomethylated, respectively, in NYGGF4-overexpression adipocytes.

**Figure 2:**
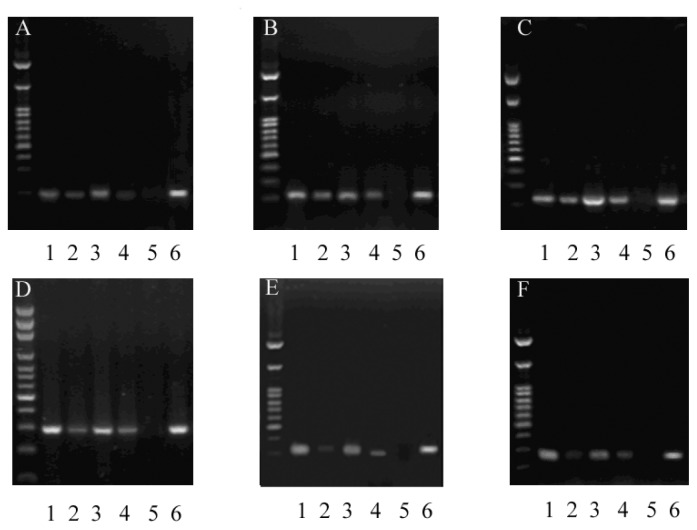
The levels of promoter methylation were measured by MSP in NYGGF4-overexpression (NYGGF4) and control adipocytes (Conl). Representative profiles of the promoter region of different genes amplified using the U and M primer combination in an agarose gel. Key: U, unmethylated; M, methylated; M+, positive control; M−, negative control. (**A**) *Cox5b*; (**B**) *Egfl9*; (**C**) *Timm23*; (**D**) *Ppp1r8*; (**E**) *Cdkn1c*; (**F**) *Pip5k1b*. Lanes: 1, NYGGF4-U; 2, NYGGF4-M; 3, Conl-U; 4, Conl-M; 5, M−; 6, M+. Marker: 2500, 1500, 1000, 900, 800, 700, 600, 500, 400, 300, 200, 100 bp.

After microarray analysis, we examined the levels of promoter methylation for several genes, to verify the microarray approach. From the genes listed in Table 1, we quantitatively measured site-specific CpG methylation upstream of *Cox5b*, *Egfl9*, *Timm23*, *Ppp1r8*, *Cdkn1c* and *Pip5k1b*. Microarray analysis predicted the promoter regions of *Cox5b*, *Egfl9* and *Timm23* to be hypermethylated in NYGGF4-overexpression adipocytes, while the promoter regions of *Ppp1r8*, *Cdkn1c* and *Pip5k1b* were predicted to be hypomethylated. MSP confirmed the site-specific CpGs of *Cox5b*, *Egfl9* and *Timm23* were hypermethylated and the site-specific CpGs of *Ppp1r8*, *Cdkn1c* and Pip5k1b were hypomethylated in NYGGF4-overexpression adipocytes. The site-specific CpG analyses were in accordance with data obtained from the microarray analysis, although the absolute differences were generally small (Figure 2).

### 2.3. Gene Ontology Analysis

**Figure 3:**
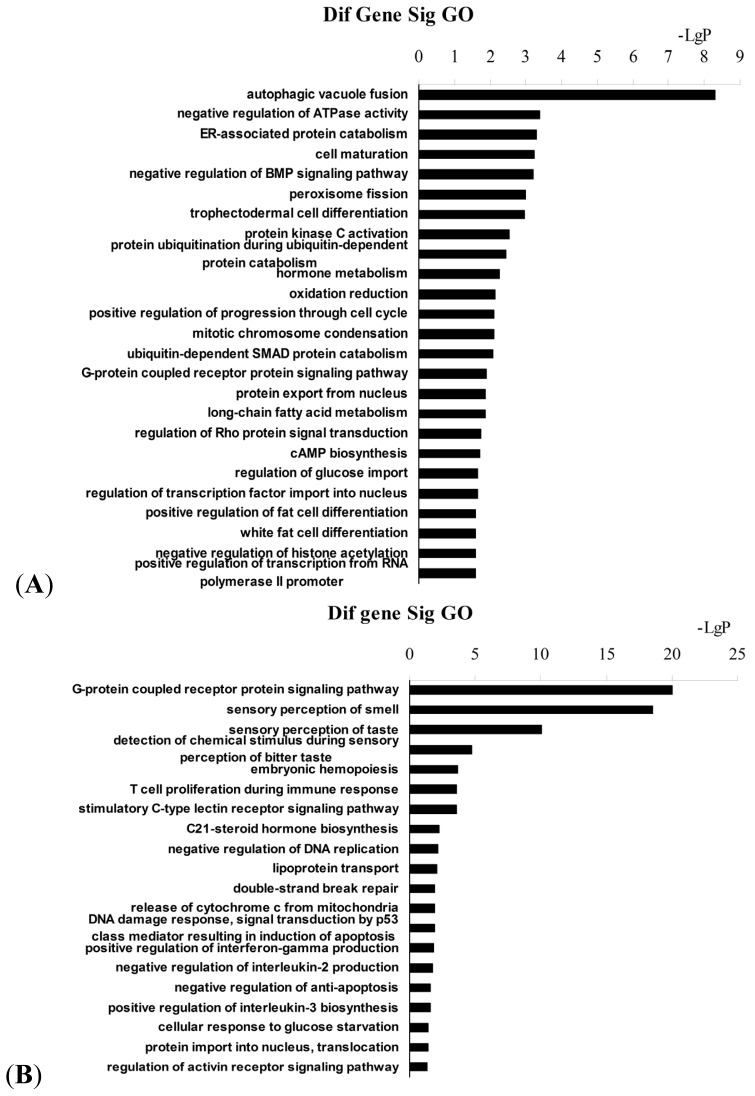
Enrichment analysis using GO terms for differentially methylated genes in NYGGF4-overexpression adipocytes. (**A**) GO terms derived from hypermethylated genes. (**B**) GO terms derived from hypomethylated genes.

Gene ontology (GO) analysis was performed to identify the molecular functions and biological processes associated with the differentially methylated genes in NYGGF4-overexpression adipocytes. The most enriched term for each group is illustrated in Figure 3; Tables S1 and S2 list all significant GO categories (*p* < 0.05). Interestingly, we observed that the genes which were hypermethylated in NYGGF4-overexpression adipocytes were potentially relevant to energy metabolism, cell differentiation and signal transduction activity, including the following categories (Figure 3A): negative regulation of ATPase activity (GO: 0032780); ER-associated protein catabolism (GO: 0030433); oxidation reduction (GO: 0055114); long-chain fatty acid metabolism (GO: 0001676); regulation of glucose import (GO: 0046324); negative regulation of the Bone morphogenetic protein (BMP) signaling pathway (GO: 0030514); ubiquitin-dependent SMAD protein catabolism (GO: 0030579); white fat cell differentiation (GO: 0050872); G-protein coupled receptor protein signaling pathway (GO: 0007186); and, regulation of Rho protein signal transduction (GO: 0035023). Meanwhile, the enriched GO terms associated with the hypomethylated genes in NYGGF4-overexpression adipocytes were involved in the following functions: (1) the G-protein coupled receptor protein signaling pathway (GO: 0007186); (2) C-type lectin receptor signaling pathway (GO: 0002223); (3) phosphatidylinositol metabolism (GO: 0046488); (4) lipoprotein transport (GO: 0042953); and, (5) regulation of interleukin (IL)-2 and 3 production (GO: 0032703; GO: 0045401 (Figure 3B)). Overall, these results suggest that these biological processes are epigenetically regulated in NYGGF4-overexpression adipocytes.

### 2.4. Pathway Analysis

**Figure 4:**
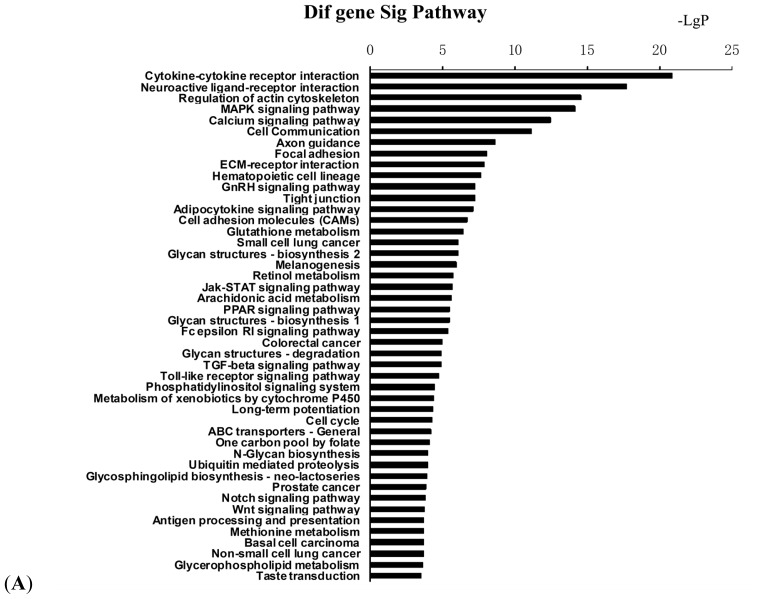

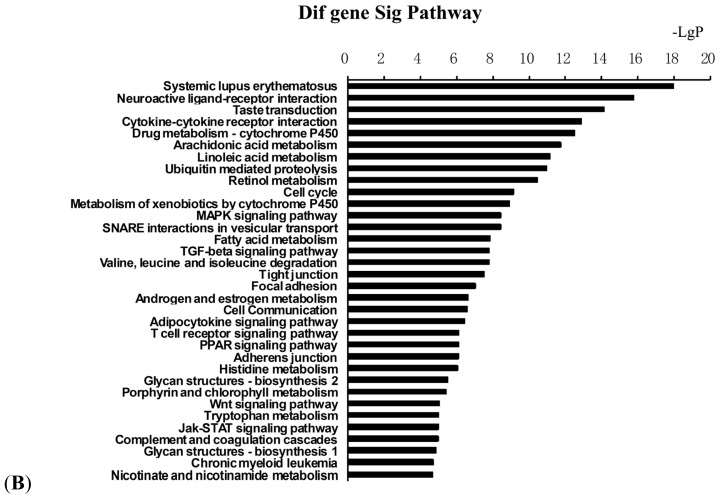
KEGG pathway enrichment analysis for the differentially methylated genes in NYGGF4-overexpression adipocytes. (**A**) KEGG pathways associated with hypermethylated genes. (**B**) KEGG pathways associated with hypomethylated genes.

**Figure 5:**
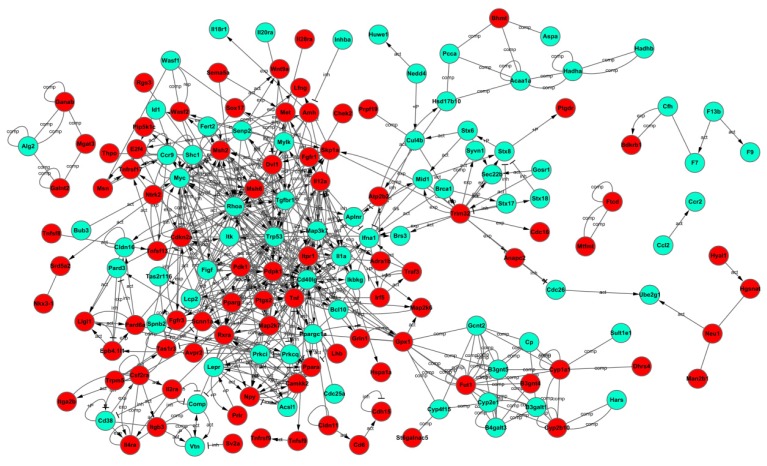
Interactions between the differentially methylated genes in NYGGF4-overexpression adipocytes, as identified using the biological network analysis function of Ingenuity Pathway Analysis. The green icons indicate hypermethylated genes; the red icons indicate hypomethylated genes in NYGGF4-overexpression adipocytes.

To further characterize the functional significance of the differentially methylated genes, we performed a systematic analysis of the genes listed in Tables S3 and S4, and searched for gene classifiers and pathways which were significantly enriched between the two adipocyte groups. More than 90 signaling pathways were detected and considered significant (*p* < 0.05), indicating that NYGGF4 affects a large number of cytokines and signaling molecules involved in a variety of signaling procedures or pathways. The biological pathways are listed in Table S2 and illustrated in Figure 4A (merged view of the pathways derived from hypermethylated genes) and Figure 4B (merged view of the pathways derived from the hypomethylated genes). Pathway analysis showed that hypermethylated genes were implicated in the following pathways: (1) cytokine–cytokine receptor interactions; (2) regulation of the actin cytoskeleton; (3) MAPK signaling pathway; (4) calcium signaling pathway; (5) adipocytokine signaling pathway; (6) retinol metabolism; (7) Jak-STAT signaling pathway; (8) PPAR signaling pathway; and, (9) phosphatidylinositol signaling system and others. The functional networks derived from the hypomethylated genes included: (1) cytokine–cytokine receptor interactions; (2) cell cycle; (3) MAPK signaling pathway; (4) fatty acid metabolism; (5) transforming growth factor-beta (TGF-β) signaling pathway; (6) adipocytokine signaling pathway; (7) Wnt signaling pathway, among others. Furthermore, we used the Kyoto Encyclopedia of Genes and Genomes(KEGG)database to create networks for these genes, according to the relationships between the genes, proteins and compounds in the database. Based on this computed signaling network, we found that multiple signaling procedures, including PPAR, MAPK and RhoA were important to the formation of this pathway network (Figure 5).

### 2.5. Discussion

In previous research, we identified that NYGGF4 was associated with obesity-associated insulin resistance; however, the underlying molecular mechanisms remained unknown. Recently, it has become evident that epigenetic factors are associated with the development of obesity [1]. In this paper, we report a genome-wide DNA methylation analysis to identify differentially methylated genes in NYGGF4-overexpression adipocytes. A total of 2018 and 3064 genes were identified to be hyper- or hypomethylated at least two-fold in NYGGF4-overexpression adipocytes. The gene ontology and KEGG pathway analysis demonstrated that these differentially methylated genes are relevant to various aspects of adipocyte biology.

The results of this study demonstrate that several of the differentially methylated genes are involved in energy metabolism. Examples include: *Tnnt1*[14] and *Tnni3*[15] (negative regulation of ATPase activity); *Slc27a1* (long-chain fatty acid metabolism) [16]; *Aspscr1* (regulation of glucose import) [17]; *Msr1* (lipoprotein transport) [18]; and, other genes associated with oxidation reduction (*Ddo*, *Cyp17a1*, *Cyp2s1*, *Cyp4f3*, *Cyp7b1*, *Epx*, *Gfer*, *Hsd17b2*, *Rdh12*, *Hsd17b6*, *Txnrd2* and *Vat1*). Other important biological processes mediated by the differentially methylated genes in NYGGF4-overexpression adipocytes include regulation of the cell cycle, fat cell differentiation, protein kinase C activation and production of IL-2 and IL-3.

Ingenuity Pathway Analysis indicated that the differentially methylated genes in NYGGF4-overexpression adipocytes were involved in multiple biological signaling pathways, including the PPAR signaling pathway, the MAPK and Rho signaling pathway and others. Peroxisome proliferator-activated receptors (PPARs) are transcription factors belonging to the nuclear receptor superfamily. Three different subtypes of PPAR (PPARα, PPARδ, PPARγ) have been identified [19]. PPARα and PPARγ are expressed in adipocytes and play important regulatory roles in adipocyte differentiation, fatty acid storage and glucose metabolism [20]. The primary function of PPARα is to regulate the transport and oxidation of free fatty acids [21]. PPARγ is known to enhance the expression of a number of genes encoding proteins involved in glucose and lipid metabolism [22]. The Ingenuity Pathway Analysis showed that the promoters of *PPARα* and *PPAR*γ were hypermethylated in NYGGF4-overexpression adipocytes, which indicates that overexpression of NYGGF4 might inhibit the expression of PPARα and PPARγ and, in turn, affect glucose metabolism in adipocytes. Further studies are warranted to investigate PPARα and PPARγ activity in NYGGF4-overexpression adipocytes.

The mitogen-activated protein kinases (MAPKs) are important mediators of signal transduction and play a key role in controlling many cellular processes, such as cell proliferation, differentiation and apoptosis [23]. More than a dozen mammalian MAPKs have been described [24]. A large body of evidence has shown that MAPK-dependent signal transduction is involved in adipocyte adipogenesis [25,26] and regulation of insulin stimuli [27]. Insulin concurrently activates both the PI3K and MAPK signaling pathways [27]. The PI3K/PKB pathway is responsible for the majority of the metabolic actions of insulin, and the MAPK pathway mediates the expression of a large number of genes and also participates in insulin-mediated glucose transport [27,28]. Intriguingly, in this study, we found that a number of molecules involved in MAPK signaling, including *Cacna2d1*, *Cacnb4*, *Cacng3*, *Fgf23*, *Fgfr1*, *Hspa1a*, *Hspa1l*, *Map2k6*, *Map2k7*, *Map4k4*, *Mapk8ip2*, *Ntrk2*, *Pla2g10*, *Pla2g6*, *Ptpn5*, *Rps6ka6*, and *Tnf*, were hypermethylated in NYGGF4-overexpression adipocytes. Additionally, other molecules including *Cacna1d*, *Cacnb3*, *Crkl*, *Ddit3*, *Dusp7*, *Fgf7*, *Ikbkg*, *Il1a*, *Map3k7*, *Myc*, *Pla2g4e*, *Ptprr*, *Tgfbr1*, and *Trp53*, were hypomethylated in NYGGF4-overexpression adipocytes. These results indicate that NYGGF4 may regulate the methylation levels of a variety of isoforms in the MAPK subfamily *via* different manners.

RhoA is a small G protein that serves as a regulator of a variety of cell functions, including migration, survival, and proliferation [29]. It is strongly suggested that RhoA is involved in insulin signaling by regulating IRS-1 and Akt [30,31]. Takaguri *et al*. [31] demonstrated that suppression of RhoA activity could inhibit the insulin-induced tyrosine phosphorylation of IRS-1 and serine/threonine phosphorylation of Akt in 3T3-L1 adipocytes. However, paradoxically, the methylation level of RhoA was reduced in NYGGF4-overexpression adipocytes. We hypothesize that this may be a compensatory effect in order to attenuate the effects of NYGGF4 on the insulin signaling pathway, or it is possible that RhoA might be involved in the regulation of some unknown biology function of NYGGF4.

## 3. Experimental Section

### 3.1. Cell Culture

3T3-L1 preadipocytes were cultured in a 6-well plate and were transfected with the NYGGF4 expression vector (pcDNA3.1Myc/His) or the empty vector as control [11]. Twenty four hours after transfection, the cells were digested and were seeded into a 96-well plate (about one cell/well). When the cell adhered, neomycin (G418) was added to the medium (800 μg/mL) to select for stably transfected cells. After two weeks of G418 addition, resistant single colonies were isolated, propagated and NYGGF4-6 × His fusion protein was identified by Western blot using an anti-6 × His antibody. The cells originating from one single clone were were grown in high glucose concentration Dulbecco’s modified Eagle’s medium (DMEM), containing 10% fetal bovine serum, and were induced to differentiate into adipocytes using a previously described method [32].

### 3.2. DNA Methylation Profiling Using the MIRA-Chip

Genomic DNA was isolated from NYGGF4-overexpression 3T3-L1 adipocytes and control cells using the QIAamp® DNA Mini Kit (Qiagen NV, Hilden, Germany). The methylated double-stranded DNA fraction was enriched by MIRA, as described previously [13,33]. Briefly, Genomic DNA was sonicated to produce random fragments (200–600 bp). Fragmented DNA is heat denatured to produce single-stranded DNA. Immunoprecipitation was performed using monoclonal antibody against 5-methylcytidine in a final volume of 500 μL IP buffer (10 mmol/L sodium phosphate, pH 7.0), 140 mmol/L NaCl, 0.05% Triton X-100) at 4 °C for 2 h. Immunoprecipitated complexes were captured with Dynabeads Protein A and M-280 sheep antimouse IgG (Roche Diagnostics GmbH, Mannheim, Germany) at 4 °C for 12 h, washed with 1 × IP buffer 4 times, treated with Proteinase K at 50 °C for 4 h, and purified by phenol-chloroform extraction and isopropanol precipitation. The immunoprecipitated methylated DNA was labeled with the fluorophore Cy5 and the control (input) genomic DNA was labeled with the fluorophore Cy3. Microarray-based DNA methylation profiling was performed according to the NimbleGen protocol (385K CpG Island plus Promoter arrays, *n* = 5).

### 3.3. Microarray Data Analysis

Signal intensity data was extracted from the scanned images of each array using NimbleScan (Roche Nimblegen, Madison, WI, USA). The ratio of the Cy5 to Cy3 signals were calculated for all high-quality hybridization dots, normalized, and transformed to the Log_2_ ratio. One-sided Kolmogorov-Smirnov (KS) tests were used to calculate the *p* value and *p* value for each probe, according to the Log_2_ Ratio for the ambient probes within a 750 bp fixed-length window. Peak scores were generated by interval analysis using a cutoff value of 2. The regions with peak scores were defined as methylated; the level of methylation positively correlated with the peak scores. If the methylation frequency of a region in NYGGF4-overexpression adipocytes was significantly higher than control cells, it was defined as hypermethylated. On the contrary, if the methylation frequency of a region in NYGGF4-overexpression adipocytes was significantly lower than control cells, it was defined as hypermethylated.

### 3.4. Bisulfite Treatment of Genomic DNA and Methylation-Specific PCR (MSP)

Genomic DNA (1 μg) from NYGGF4-overexpression adipocytes and control cells DNA was bisulfite-treated using the EpiTect Bisulfite Kit (Qiagen, NV, Hilden, Germany) according to the manufacturer’s protocol, to convert unmethylated cytosine residues to uracil, and eluted into 10 μL buffer. MSP was performed using methylation-specific primers (Table 1) specific for methylated and unmethylated sequences using Hot-Star *Taq* DNA polymerase (Tiangen, Beijing, China) and the MSP products were visualized under UV light after electrophoresis on 3.5% agarose gels containing ethidium bromide.

### 3.5. Gene-Ontology Analysis

GO analysis was used to analyze the main functions of the differentially expressed genes, according to the GO database which provides the key functional classifications for the National Center for Biotechnology Information (NCBI) [34]. Generally, Fisher’s exact test and the Chi-square test were used to classify the GO categories, and the false discovery rate (FDR) [35] was calculated to correct the *p* values; the smaller the FDR, the smaller the error in judging the *p* value. We computed the *p* values for the GOs of all differentially methylated genes. Enrichment provides a measure of the significance of the function: as enrichment increases, the corresponding function is more specific, which helps to identify GOs with more concrete functional descriptions. Within the significant category, enrichment *R*e was given by:

(1) $$R\text{e}=(n_{f}/n)/(N_{f}/N)$$

where *n_f_* is the number of differentially methylated genes within the particular category, *n* is the total number of genes within the same category, *N_f_* is the number of differentially methylated genes in the entire microarray, and *N* is the total number of genes in the microarray [36].

### 3.6. Ingenuity Pathway Analysis (IPA)

Ingenuity Pathway Analysis was used to identify the significant pathways for the differentially methylated genes according to the KEGG, Biocarta and Reatome databases. The Fisher’s exact test and Chi-square test were used to select significant pathways; the threshold of significance was defined by the *p* value and FDR. Enrichment (*R*e) was calculated as previously described [37].

### 3.7. Statistical Analysis

Differences in GO terms and pathway analysis between the hypermethylated or hypomethylated genes in NYGGF4-overexpression adipocytes were assessed using the Chi-squared test or Fisher’s exact test. Two-sided probability values of <0.05 were considered statistically significant.

## 4. Conclusions

In conclusion, the study provides valuable insight into methylation changes in NYGGF4-overexpression adipocytes, and offers clues for exploration of the mechanisms by which NYGGF4 regulates insulin sensitivity. Further functional studies may provide additional insight into role of these differentially methylated genes in NYGGF4-induced insulin resistance.

## Figures and Tables

**Table 1:** Primer sequences for the methylated and unmethylated sequences

Gene	Forward primer (5′-3′)	Reverse primer (5′-3′)	Product size (bp)
*Cox5b* unmeth	TTAAAGTTGTTTGTAGTT	AAAACAACACTAAAAATA	187
*Cox5b* meth	AAAGTTGTTTGTAGTTT	GAAACAACGCTAAAAATA	187
*Egfl9* unmeth	ATTTTGAGATGTGGAGT	CAACTCTCCTATTTTAACC	347
*Egfl9* meth	TCGAGATGTGGAGTTGT	AAAACATATACTCAACCAT	344
*Timm23* unmeth	TTTTATTTATTAATTTGGA	AAAACATATACTCAACCAT	219
*Timm23*meth	TATTTATTAATTCGGATA	ACGTATACTCGACCGTAAC	219
*Ppp1r8* unmeth	TTGAATTAGTGGTTTTAT	CCATTTAACAAATCTAAAA	216
*Ppp1r8* meth	GAATTAGTGGTTTTATTC	ATTTAACGAATCTAAAACA	216
*Cdkn1c* unmeth	TTTTGTTTGTAGATAAAG	CCTACCTATTCACTTACTC	327
*Cdkn1c* meth	GCGGTGTTACGTTATCGT	ACAACGCACTCGACCTAT	327
*Pip5k1b* unmeth	GTATTGATTAGAGTGTTA	ACTACAAATATACAATACA	263
*Pip5k1b* meth	TATCGATTAGAGCGTTAG	CTACGAATATACAATACGC	263
